# Periprosthetic Distal Femoral Fracture Following Total Knee Arthroplasty Associated With Anterior Femoral Notching: A Case Report

**DOI:** 10.7759/cureus.106761

**Published:** 2026-04-10

**Authors:** Nay Aung Zin, Thiha Zaw, Sandar Shune Let Aung, Min Maung Maung, Htoo Aung Naing Shwe, Tun Tun

**Affiliations:** 1 Orthopaedics and Traumatology, Kulhudhuffushi Regional Hospital, Kulhudhuffushi, MDV; 2 Orthopaedics and Traumatology, Mediland UHC Hospital, Yangon, MMR; 3 Anaesthesiology, Indira Gandhi Memorial Hospital, Malé, MDV; 4 Orthopaedics and Trauma, Central Clinic, Malé, MDV; 5 Orthopaedics and Trauma, Himmafushi Health Center, Himmafushi, MDV; 6 Orthopaedics and Trauma, University of Medicine, Mandalay, Mandalay, MMR

**Keywords:** bilateral knee surgery, early postoperative complications, femoral notching, fracture fixation, joint replacement complications, notching and fracture risk, orthopedic surgery, peri-prosthesis fracture, rehabilitation in tkr, total knee replacement

## Abstract

Periprosthetic distal femoral fractures are uncommon but potentially serious complications following total knee arthroplasty (TKA). Several patient-related and surgical factors contribute to their occurrence, including osteoporosis, trauma, implant malalignment, and technical factors such as anterior femoral notching. Femoral notching, caused by violation of the anterior femoral cortex during femoral preparation, has been suggested to weaken the structural integrity of the distal femur and increase susceptibility to fracture. However, the association between femoral notching and periprosthetic fracture remains controversial.

We report the case of a 47-year-old woman who developed a periprosthetic distal femoral fracture following bilateral TKA. Radiographic evaluation demonstrated a supracondylar femoral fracture adjacent to the femoral component with evidence of anterior femoral notching. The patient presented with acute knee pain and functional limitation during early postoperative rehabilitation. Surgical fixation was performed, followed by a structured rehabilitation program.

This case highlights the potential biomechanical implications of anterior femoral notching during TKA. Although periprosthetic fractures are multifactorial in origin, cortical violation may act as a stress riser that predisposes the distal femur to fracture. Careful surgical technique and vigilant postoperative monitoring may help reduce the risk of this complication.

## Introduction

Total knee arthroplasty (TKA) is a widely performed surgical procedure for the management of end-stage knee osteoarthritis and has been shown to provide significant pain relief and improved functional outcomes. Despite its high success rate, postoperative complications may occur. Among these, periprosthetic distal femoral fractures represent a relatively rare but serious complication that can lead to significant morbidity and often require complex surgical management [1,2].

Several risk factors have been associated with periprosthetic femoral fractures following TKA, including osteoporosis, advanced age, female sex, chronic steroid use, neurological disorders, and technical factors related to the surgical procedure [3,4]. One surgical factor that has received considerable attention is anterior femoral notching, which occurs when the anterior cortex of the distal femur is inadvertently violated during femoral component preparation [5].

Femoral notching has been hypothesized to weaken the structural integrity of the distal femur by creating a stress concentration point, thereby predisposing the bone to fracture. Although some clinical studies report no significant association between femoral notching and periprosthetic fracture risk, other studies suggest that deeper notching may significantly increase the risk of supracondylar femoral fractures following TKA [5-8].

In this report, we present a case of a periprosthetic distal femoral fracture following bilateral TKA in a 47-year-old female patient, highlighting the potential biomechanical implications of anterior femoral notching in the development of postoperative fractures.

## Case presentation

A 47-year-old woman presented with severe bilateral knee pain and progressive stiffness that significantly impaired her ability to perform daily activities and weight-bearing. The symptoms had progressively worsened over several years despite conservative management, including physiotherapy and intra-articular corticosteroid injections. Due to persistent pain and functional limitation, the patient elected to undergo bilateral total knee arthroplasty (TKA).

**Figure 1:**
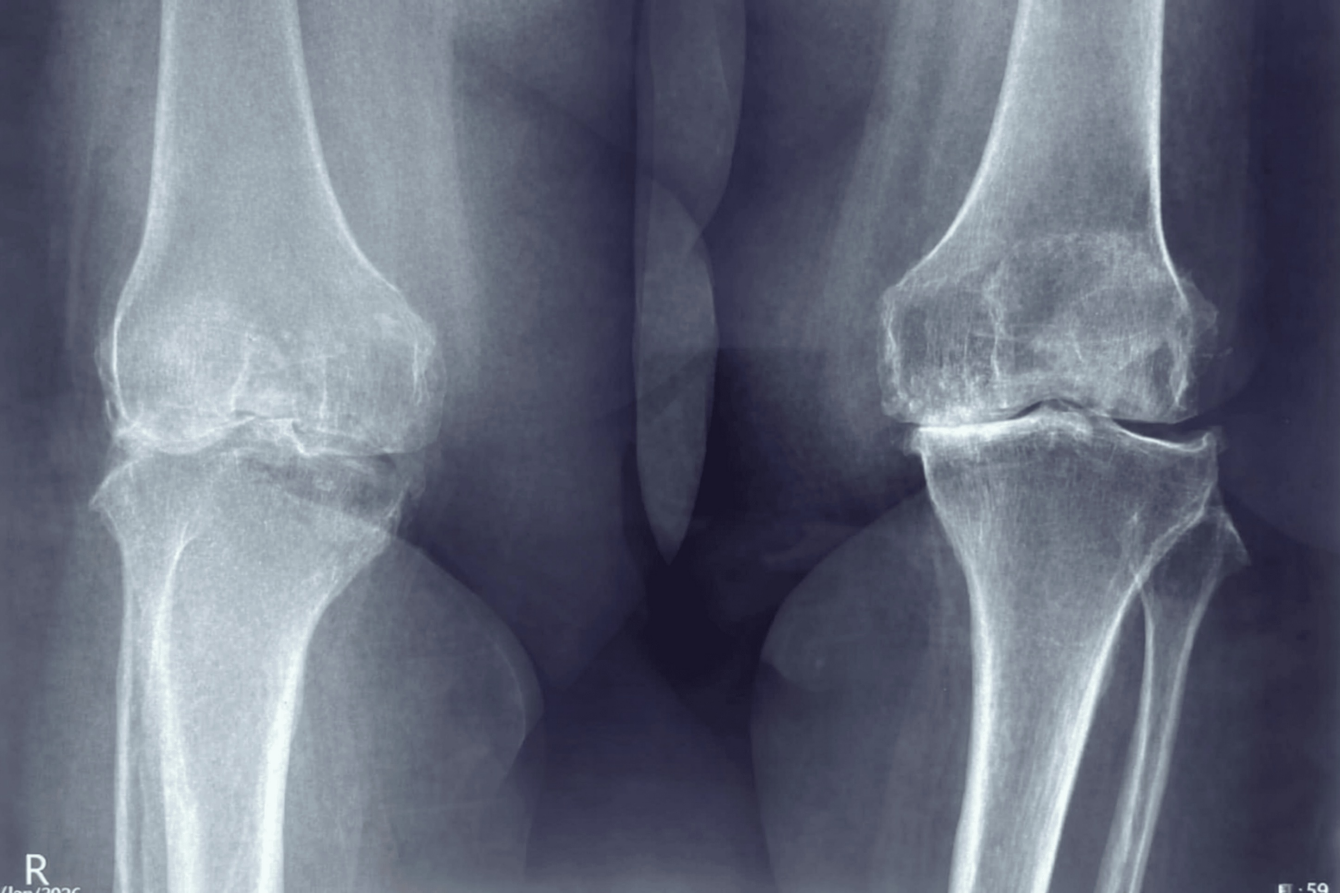
Preoperative standing anteroposterior radiograph of both knees X-ray demonstrating advanced degenerative osteoarthritis characterized by joint space narrowing, osteophyte formation, and subchondral sclerosis.

**Figure 2:**
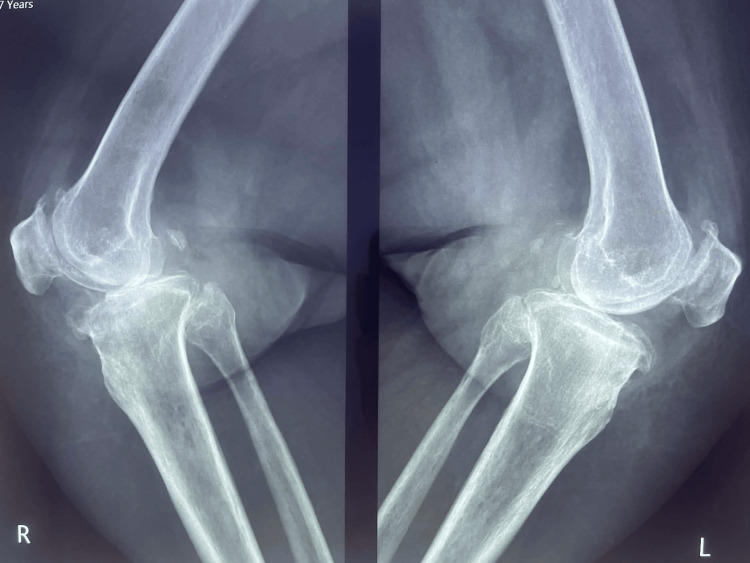
Preoperative lateral radiographs of both knees Preoperative lateral radiographs of both knees showing severe degenerative joint changes consistent with end-stage osteoarthritis.

The patient was 5 feet 3 inches tall and weighed 220 lbs, corresponding to a body mass index (BMI) of 39 kg/m². She had no significant endocrine disorders or other chronic systemic diseases. Preoperative standing radiographs of both knees demonstrated advanced degenerative changes consistent with end-stage osteoarthritis, including joint space narrowing, subchondral sclerosis, and osteophyte formation. Additional lateral radiographs confirmed severe degenerative joint disease involving both knees (Figures 1-2).

**Figure 3:**
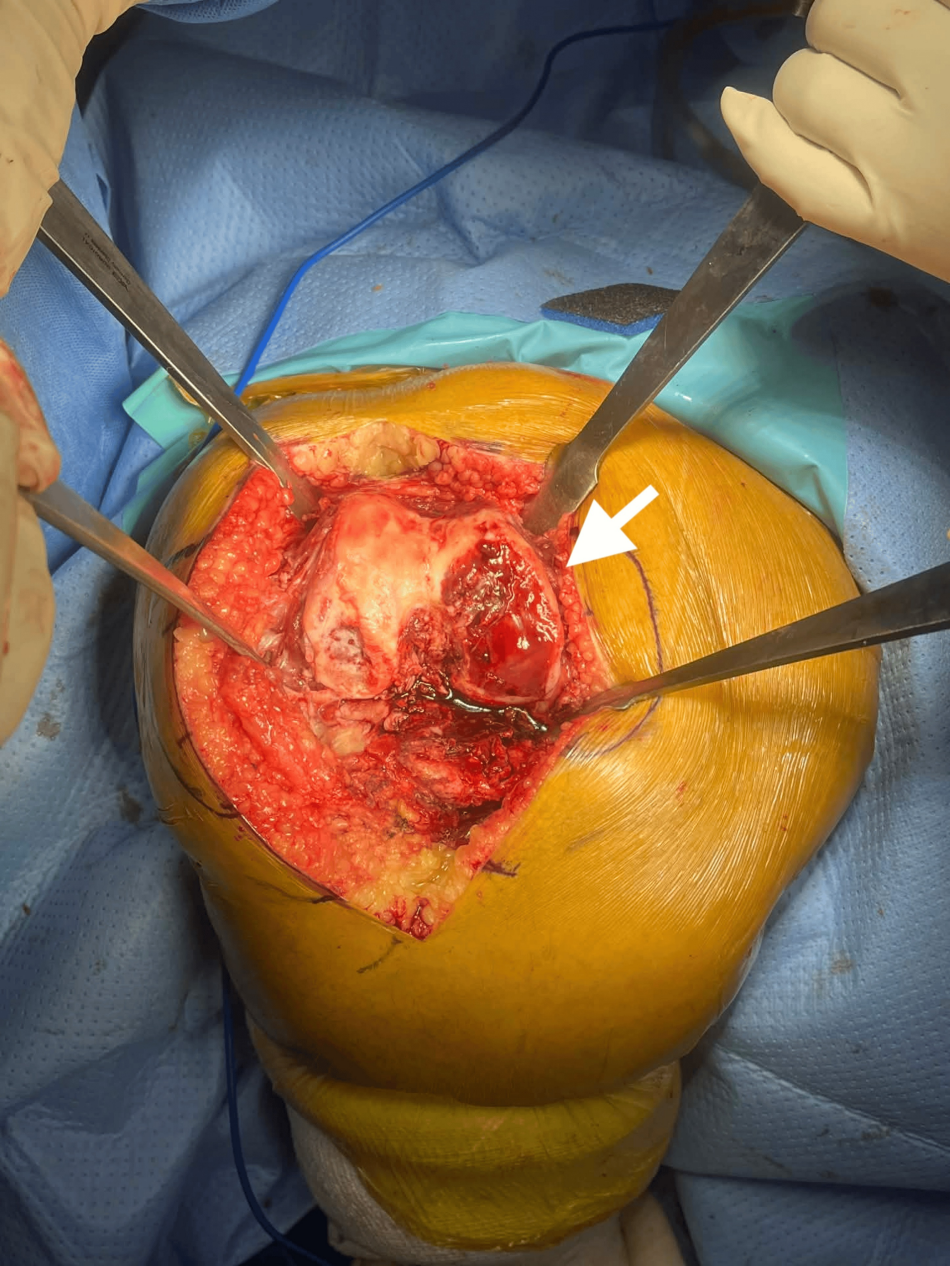
Intraoperative photography Intraoperative photograph of the distal femur during total knee arthroplasty demonstrating exposure of the femoral condyles. The arrow indicates severe destruction of the articular cartilage over the medial femoral condyle consistent with advanced osteoarthritis.

The patient subsequently underwent bilateral TKA. Intraoperatively, the distal femur was exposed using a standard surgical approach, and preparation of the femoral condyles was performed prior to placement of the prosthetic components (Figure 3).

**Figure 4:**
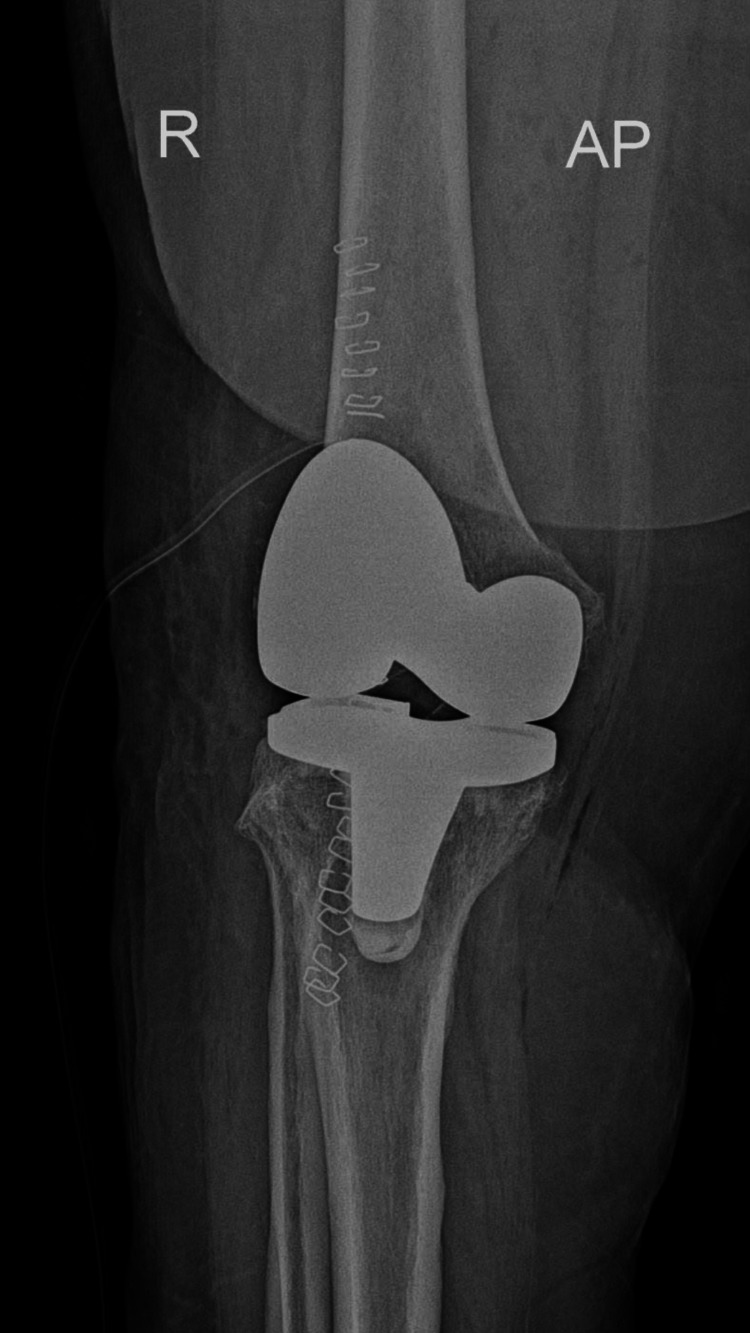
Immediate postoperative anteroposterior radiograph of the right knee Immediate postoperative anteroposterior radiograph of the right knee demonstrating satisfactory positioning and alignment of the femoral and tibial components following total knee arthroplasty.

**Figure 5:**
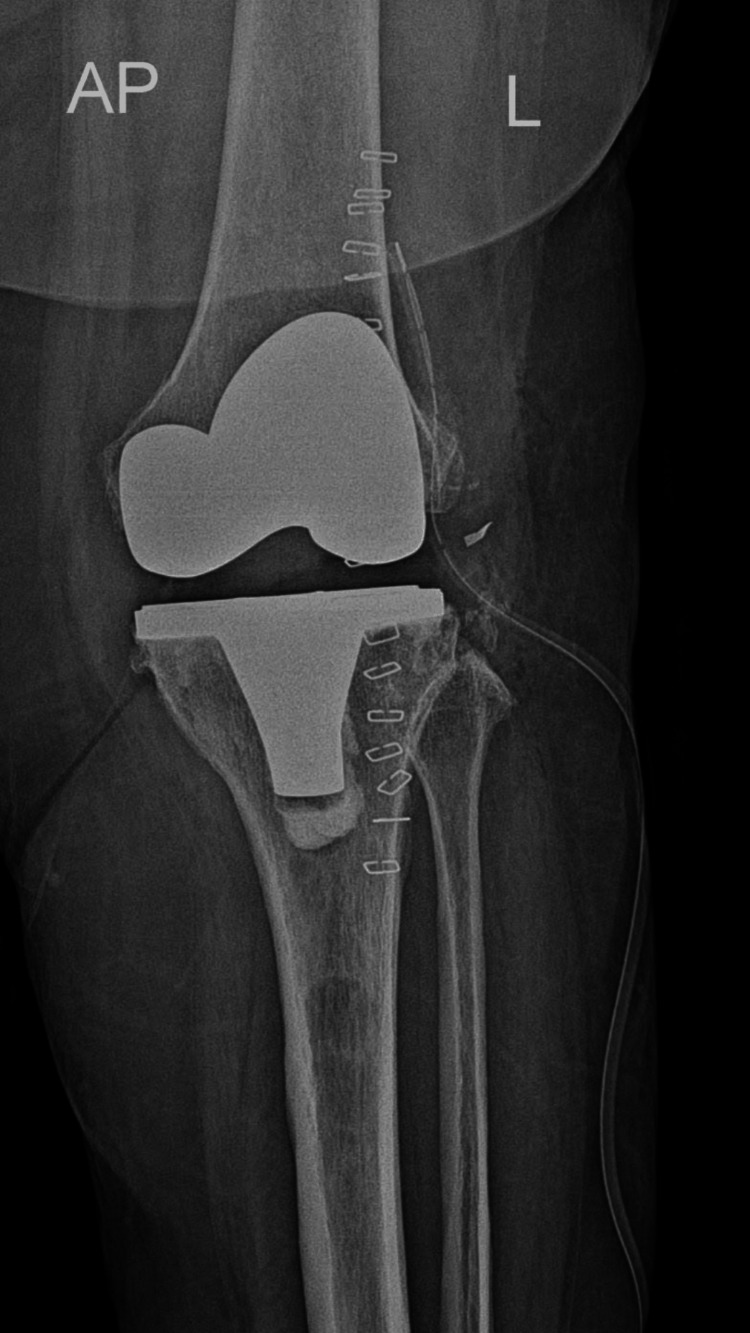
Immediate postoperative anteroposterior radiograph of the left knee Immediate postoperative anteroposterior radiograph of the left knee demonstrating appropriate alignment and positioning of the prosthetic components.

The procedure was completed without immediate intraoperative complications. Immediate postoperative radiographs were obtained to evaluate prosthetic positioning. The anteroposterior views demonstrated satisfactory alignment and positioning of the femoral and tibial components in both knees (Figures 4-5).

**Figure 6:**
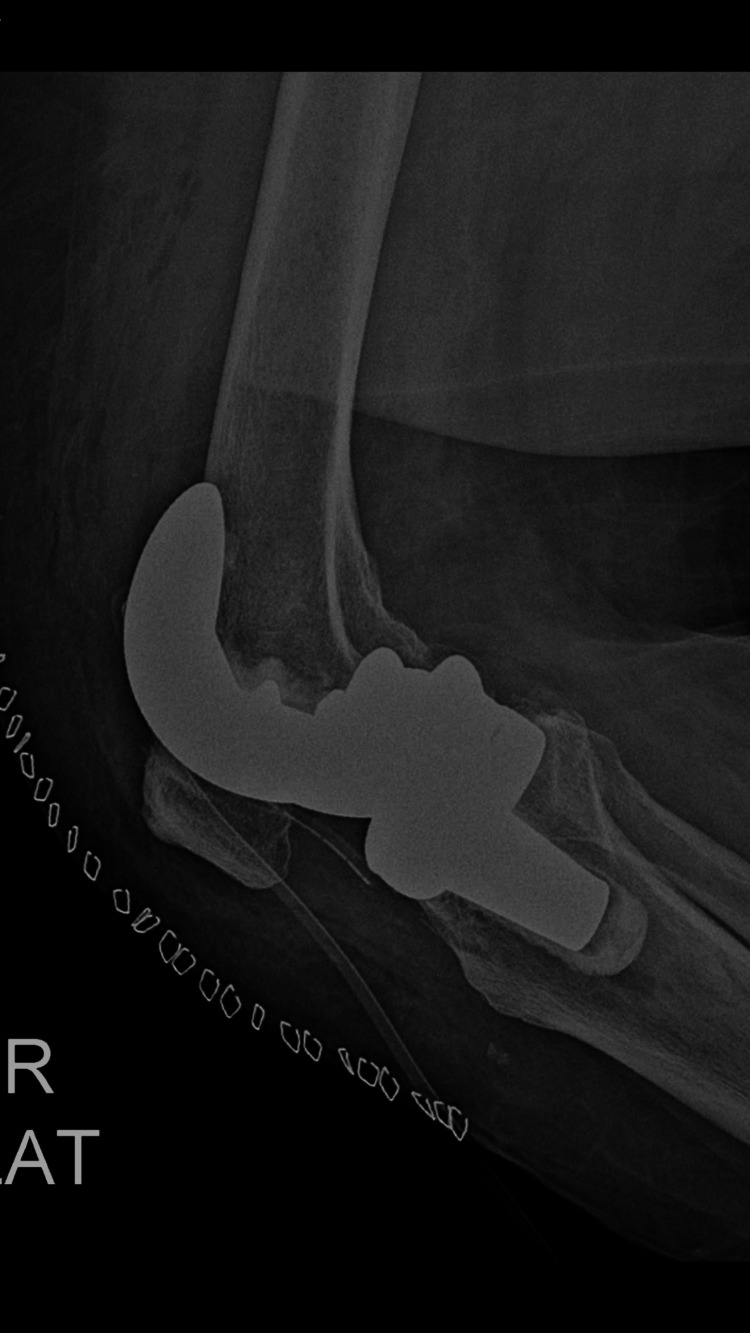
Immediate postoperative lateral radiograph of the right knee Immediate postoperative lateral radiograph of the right knee confirming appropriate placement and alignment of the femoral component following total knee arthroplasty.

**Figure 7:**
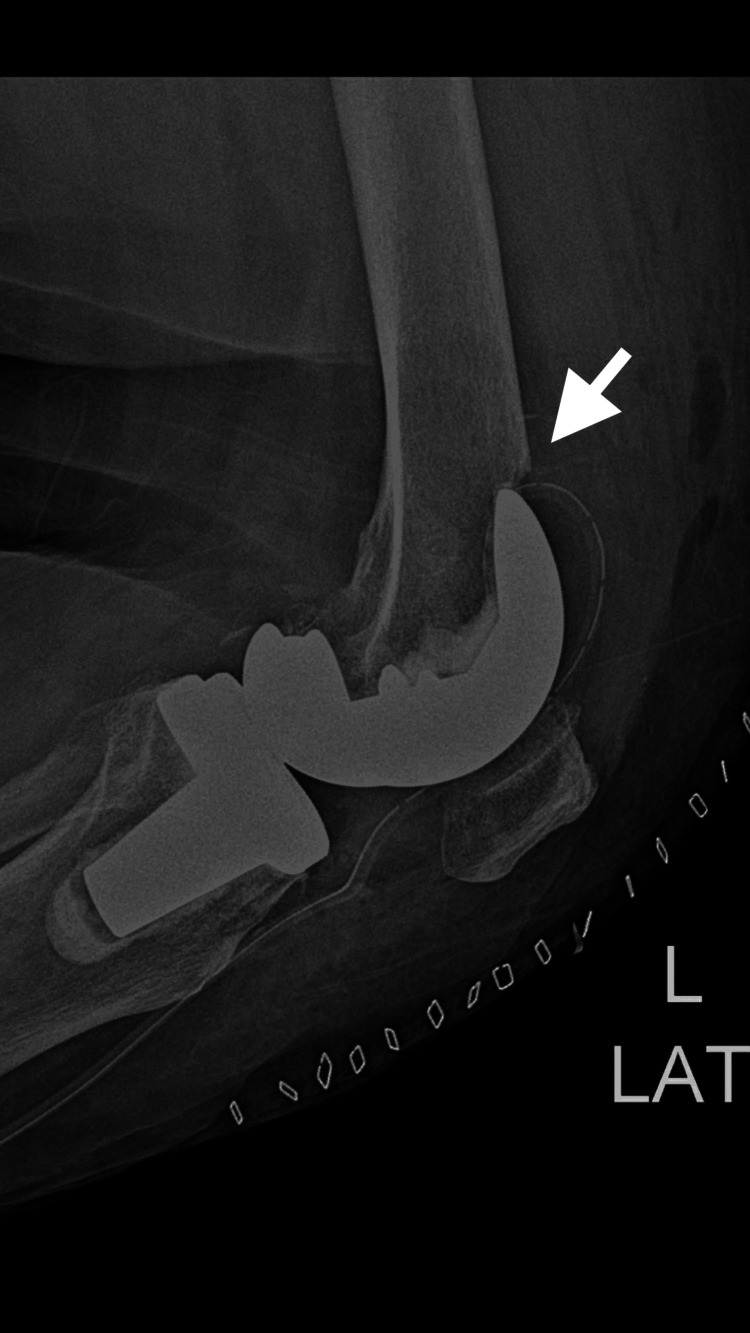
Immediate postoperative lateral radiograph of the left knee Immediate postoperative lateral radiograph of the left knee demonstrating anterior femoral cortical notching adjacent to the femoral component, which may represent a potential biomechanical stress riser.

Postoperative lateral radiographs of the right knee confirmed appropriate prosthetic alignment without complications (Figure 6). However, the lateral radiograph of the left knee demonstrated anterior femoral cortical notching at the distal femur adjacent to the femoral component (Figure 7). This cortical violation was noted as a potential structural weakness of the anterior femoral cortex.

**Figure 8:**
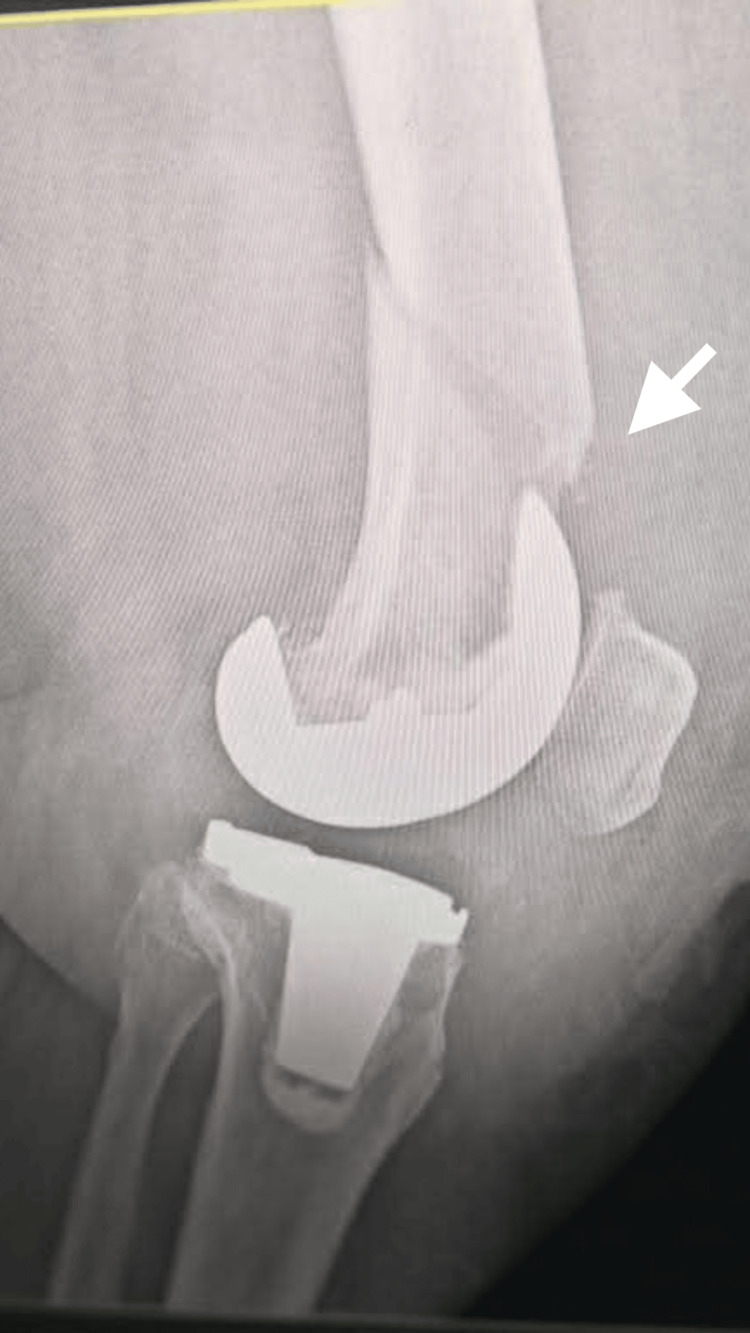
Lateral radiograph of the left knee Lateral radiograph of the left knee demonstrating a supracondylar periprosthetic distal femoral fracture above the femoral component following total knee arthroplasty. The arrow indicates the fracture line proximal to the femoral prosthesis.

**Figure 9:**
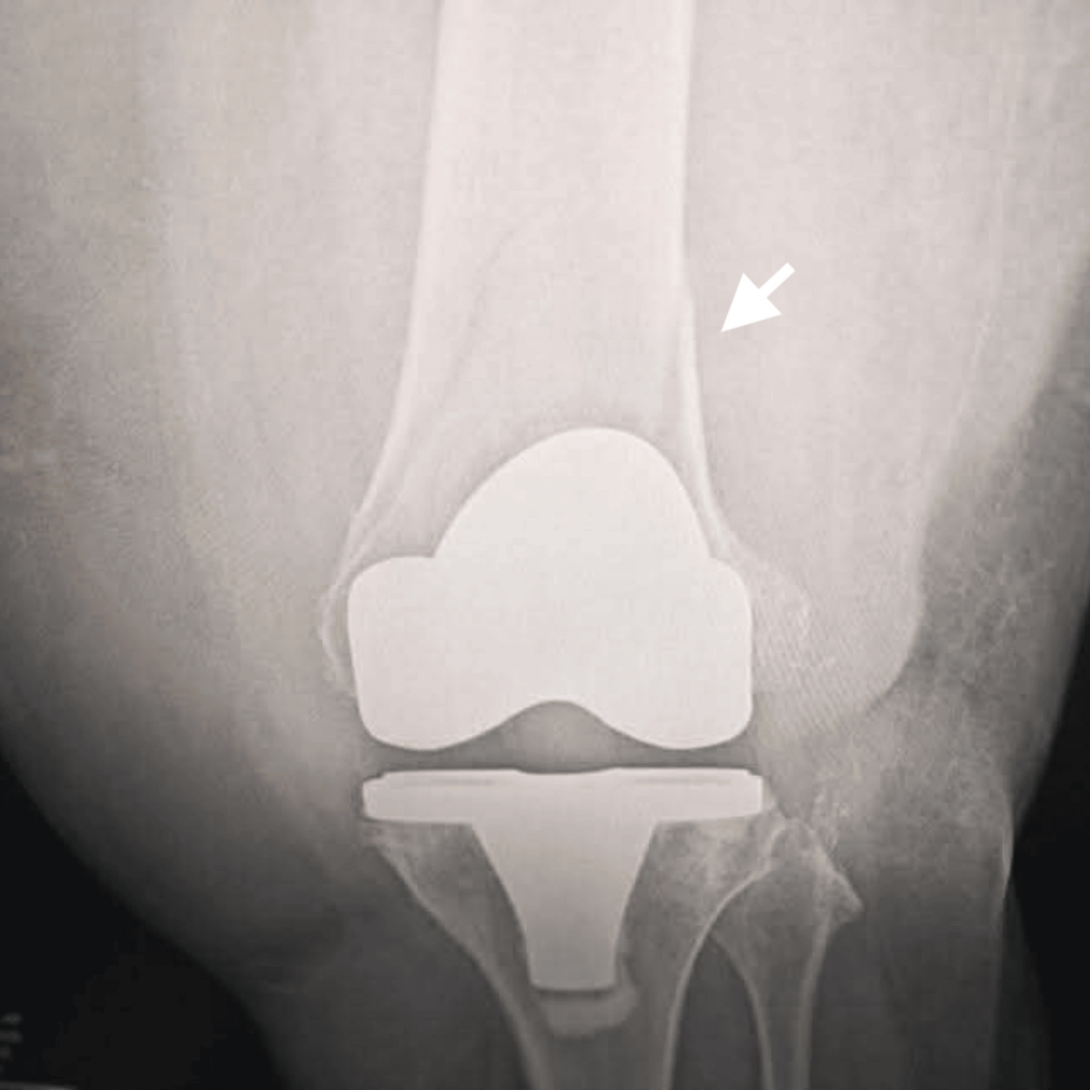
Anteroposterior radiograph of the left knee Anteroposterior radiograph of the left knee showing a displaced supracondylar periprosthetic distal femoral fracture adjacent to the femoral component of the total knee arthroplasty. The arrow highlights the fracture site above the prosthetic component.

On postoperative day 25, while performing rehabilitation exercises, the patient experienced a sudden onset of severe pain in the left knee accompanied by difficulty bearing weight. Radiographic evaluation revealed a supracondylar periprosthetic distal femoral fracture located proximal to the femoral component (Figures 8-9).

According to the Lewis and Rorabeck classification, the fracture was consistent with a Type II periprosthetic distal femoral fracture, characterized by displacement with a stable prosthetic component. The fracture occurred in close proximity to the previously identified anterior femoral notch, suggesting a possible relationship between cortical weakening and fracture development.

**Figure 10:**
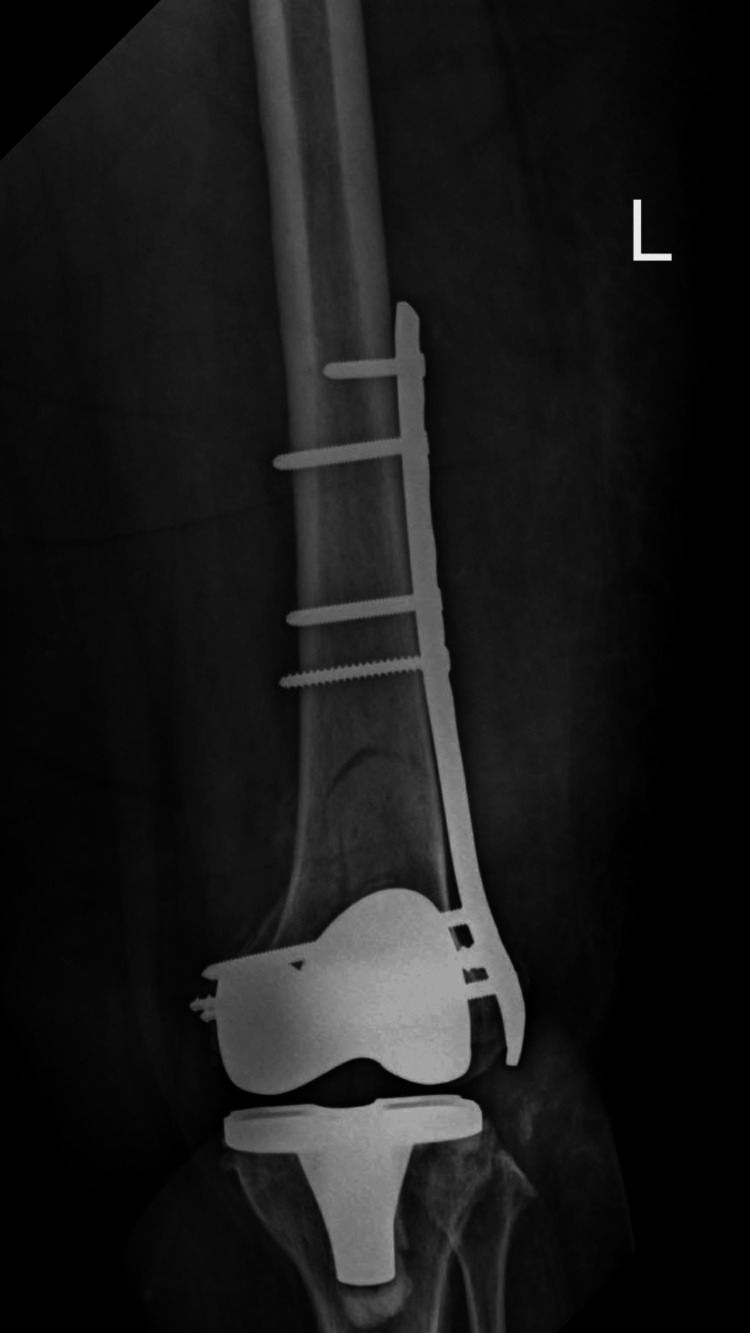
Anteroposterior radiograph of the left femur following surgical fixation of a periprosthetic distal femoral fracture. The image demonstrates a distal femoral locking plate with multiple screws providing stable fixation proximal to the femoral component of the total knee arthroplasty.

**Figure 11:**
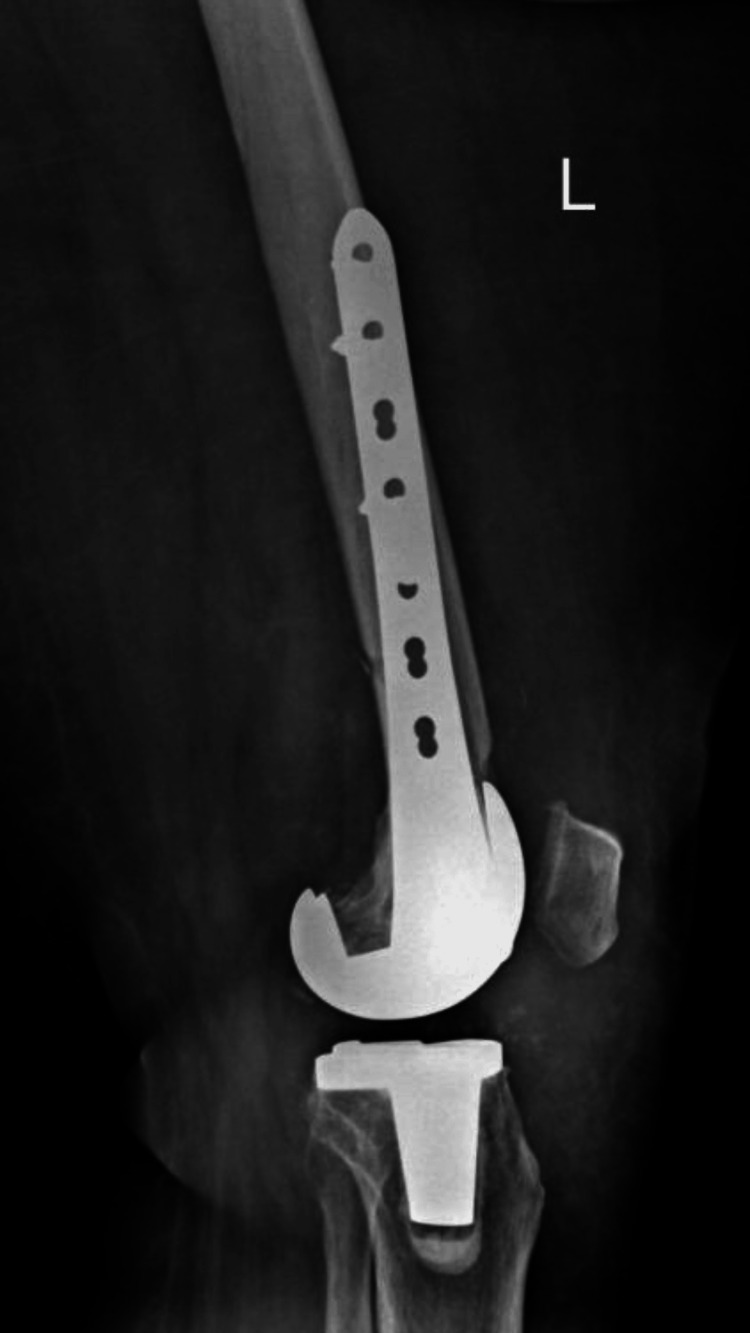
Lateral radiograph of the left femur demonstrating postoperative fixation of the periprosthetic distal femoral fracture using a locking plate system. The prosthetic components remain well aligned, and fixation appears stable without evidence of hardware failure.

Following diagnosis, the patient underwent surgical fixation of the periprosthetic distal femoral fracture to restore mechanical stability and maintain prosthetic integrity. Postoperative radiographs demonstrated stabilization of the fracture with maintained prosthetic alignment (Figures 10-11).

Postoperative management included controlled weight-bearing, a structured physiotherapy program, and serial radiographic monitoring to assess fracture healing and prosthetic stability. Follow-up radiographs obtained on postoperative day 30 demonstrated early signs of fracture healing without displacement, and the patient continued rehabilitation under close clinical supervision.

## Discussion

Periprosthetic distal femoral fractures are uncommon but clinically important complications following total knee arthroplasty (TKA). They have been reported to occur in approximately 0.3% to 2.5% of primary TKAs. This is the less significant occurrence, but we need to be careful as it might require complex management because the treatment must preserve prosthetic stability while achieving fracture fixation and early mobilization [1,2].

These fractures are commonly classified according to the Lewis and Rorabeck classification system, which is based on fracture displacement and prosthetic stability [3]. In this system, Type I fractures are nondisplaced with a stable prosthesis, Type II fractures are displaced with a stable prosthesis, and Type III fractures are associated with a loose prosthetic component. In the present case, the fracture was consistent with a Type II periprosthetic distal femoral fracture.

Multiple patient-related and surgical factors have been associated with an increased risk of periprosthetic fracture after TKA. Reported risk factors include advanced age, osteoporosis, female sex, revision arthroplasty, neurological comorbidity, and technical factors related to the index procedure [1,2,4]. In the present case, obesity may also have contributed to increased mechanical loading across the distal femur during the early postoperative period.

One technical factor of particular interest is anterior femoral notching, which occurs when the anterior femoral cortex is violated during femoral preparation. Management options for periprosthetic distal femoral fractures include conservative treatment, locking plate fixation, retrograde intramedullary nailing, and distal femoral replacement, depending on fracture characteristics, bone quality, and prosthetic stability. Biomechanical studies suggest that such notching may weaken the distal femur by creating a stress riser and increasing local stress concentration [5,6]. Zalzal et al. demonstrated that larger and sharper notches, particularly those close to the implant, increase local stress within the distal femur [5]. Gujarathi et al. likewise reported that anterior femoral notching may predispose the femur to supracondylar fracture, although fractures remain relatively uncommon overall [6].

Clinical studies evaluating the relationship between anterior femoral notching and periprosthetic fracture have yielded mixed findings. Puranik et al. found no significant correlation between femoral notching and supracondylar fracture following TKA in their prospective series, although they still recommended that notching should be avoided [7]. In contrast, the systematic review and meta-analysis by Stamiris et al. demonstrated that anterior femoral notching of 3 mm or greater is associated with a significantly increased risk of supracondylar periprosthetic femoral fracture after TKA [8].

Compared with previously reported cases, most periprosthetic distal femoral fractures occur several months to years after TKA, often following minor trauma or progressive bone weakening. In contrast, the present case occurred within 25 days of surgery, representing an unusually early presentation. This early occurrence suggests a combination of mechanical vulnerability due to anterior femoral notching and increased loading associated with obesity during rehabilitation [1].

Regarding management, treatment options for periprosthetic distal femoral fractures include conservative management, locking plate fixation, retrograde intramedullary nailing, and distal femoral replacement, depending on fracture pattern, bone quality, and prosthetic stability. In this case, locking plate fixation was selected as the prosthesis was stable and allowed preservation of the existing implant while achieving stable fixation and early mobilization.

In the present case, postoperative lateral radiographs demonstrated anterior femoral cortical notching, and the subsequent supracondylar periprosthetic fracture developed at that level during rehabilitation. Although causation cannot be established from a single case, the temporal and radiographic association suggests that cortical weakening from notching may have contributed to fracture development in the setting of early postoperative loading.

This case highlights the importance of meticulous femoral preparation during TKA to avoid anterior cortical violation. It also underscores the need for careful postoperative monitoring and cautious rehabilitation in patients with recognized risk factors for periprosthetic fracture.

## Conclusions

Periprosthetic distal femoral fractures are rare but potentially serious complications following total knee arthroplasty. Anterior femoral notching may increase fracture risk by weakening the structural integrity of the distal femur and creating a stress concentration point.

This case highlights the importance of meticulous surgical technique to avoid anterior femoral cortical violation during femoral preparation. Careful postoperative monitoring and appropriately guided rehabilitation may help reduce the risk of periprosthetic fracture and improve patient outcomes.
